# Supplementary material for: A facile strategy for realizing room temperature phosphorescence and single molecule white light emission
Source: Nat Commun. 2018 Jul 27;9:2963. doi: 10.1038/s41467-018-05298-y (PMC6063922; doi:10.1038/s41467-018-05298-y)
Supplement: Supplementary file 1 — Supplementary Information [file 41467_2018_5298_MOESM1_ESM.pdf]

A facile strategy for realizing room temperature phosphorescence and single molecule white light emission

Wang et al.

A facile strategy for realizing room temperature phosphorescence and single molecule white light emission

Jianguo Wang[1,3], Xinggui Gu[1,2,*], Huili Ma[4], Qian Peng[5], Xiaobo Huang[6], Xiaoyan Zheng[1], Simon H. P. Sung[1], Guogang Shan[1], Zhigang Shuai[4], Jacky W. Y. Lam[1] & Ben Zhong Tang[1,7,8,*]

[1]Department of Chemistry, Hong Kong Branch of Chinese National Engineering, Research Center for Tissue Restoration and Reconstruction, Institute of Molecular Functional Materials, State Key Laboratory of Molecular Nanoscience, Division of Life Science and Biomedical Engineering, The Hong Kong University of Science and Technology, Clear Water Bay, Kowloon, Hong Kong, China.

[2]Beijing Advanced Innovation Center for Soft Matter Science and Engineering, Beijing University of Chemical Technology, Beijing 100029, China.

[3]Key Laboratory of Organo-Pharmaceutical Chemistry, Gannan Normal University, Ganzhou 341000, China.

[4]Key Laboratory of Organic Optoelectronics and Molecular Engineering, Department of Chemistry, Tsinghua University, Beijing 100084, China.

[5]Key Laboratory of Organic Solids, Beijing National Laboratory for Molecular Science, Institute of Chemistry, Chinese Academy of Sciences, Beijing 100190, China.

[6]College of Chemistry and Materials Engineering, Wenzhou University, Wenzhou 325035, China.

[7]NSFC Center for Luminescence from Molecular Aggregates, SCUT-HKUST Joint Research Institute, State Key Laboratory of Luminescent Materials and Devices, South China University of Technology, Guangzhou 510640, China.

[8]HKUST-Shenzhen Research Institute, No. 9 Yuexing 1st RD, South Area, Hi-tech Park, Nanshan, Shenzhen 518057, China.

[*]Correspondence and requests for materials should be addressed to X.G. (email: guxinggui@mail.buct.edu.cn) or to B.Z.T. (email: tangbenz@ust.hk)

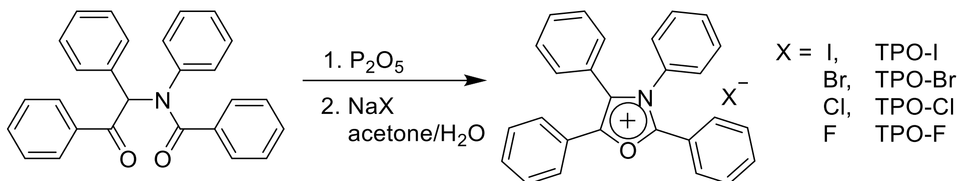

3

Supplementary Figure 1 Synthesis route of TPO-I, TPO-Br, TPO-Cl, and TPO-F.

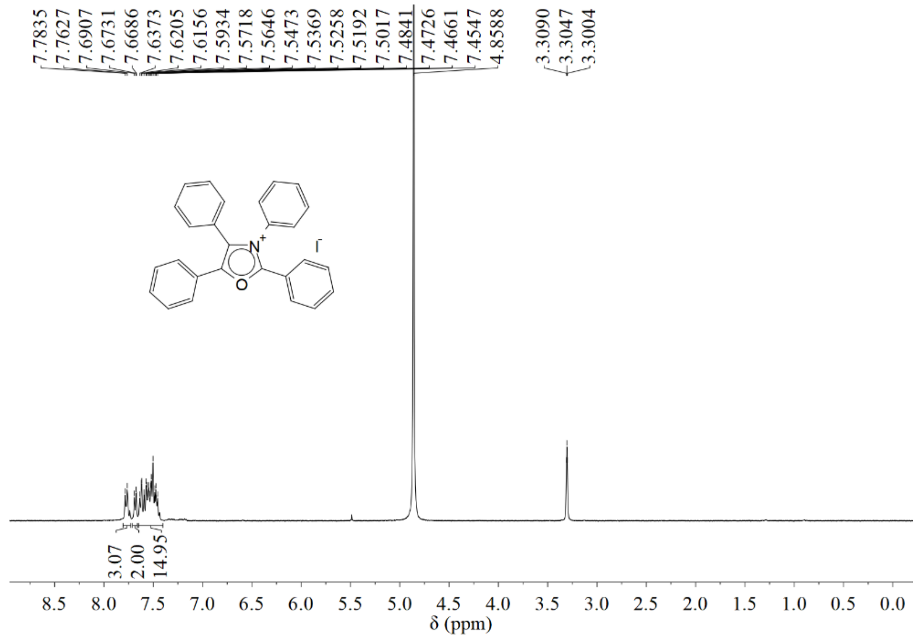

Supplementary Figure 2 ^{1}H NMR spectrum of TPO-I in CD_3OD.

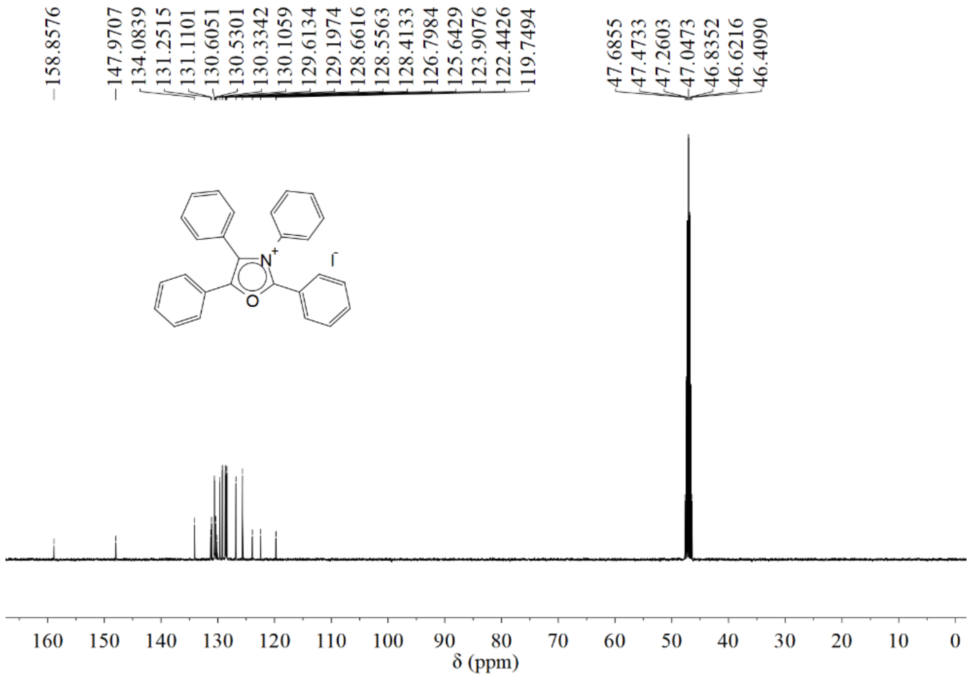

Supplementary Figure 3 ^{13}C NMR spectrum of TPO-I in CD_3OD.

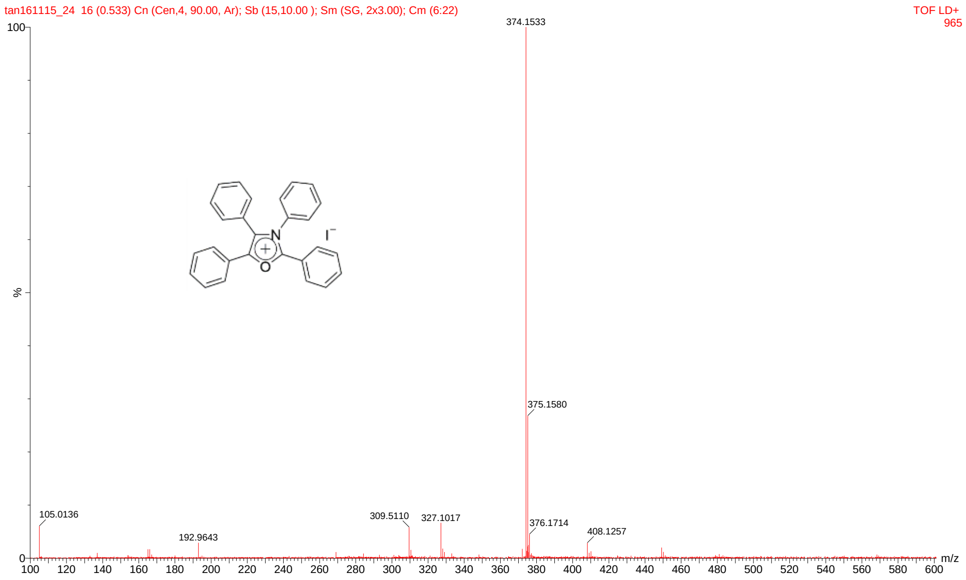

Supplementary Figure 4 HRMS spectrum of TPO-I.

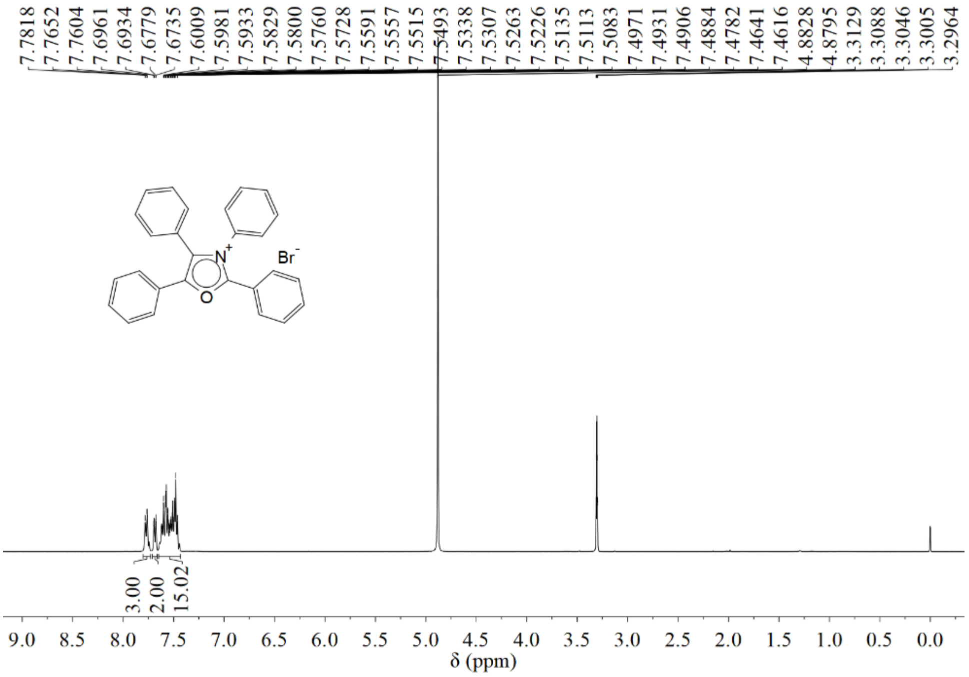

Supplementary Figure 5 ^{1}H NMR spectrum of TPO-Br in CD_3OD.

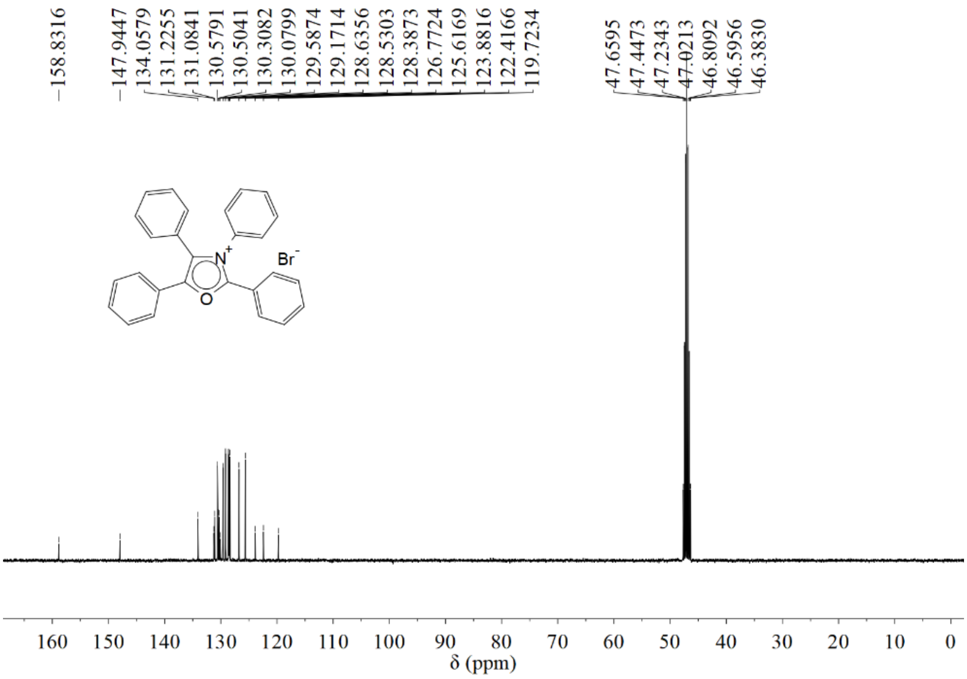

Supplementary Figure 6 ^{13}C NMR spectrum of TPO-Br in CD_3OD.

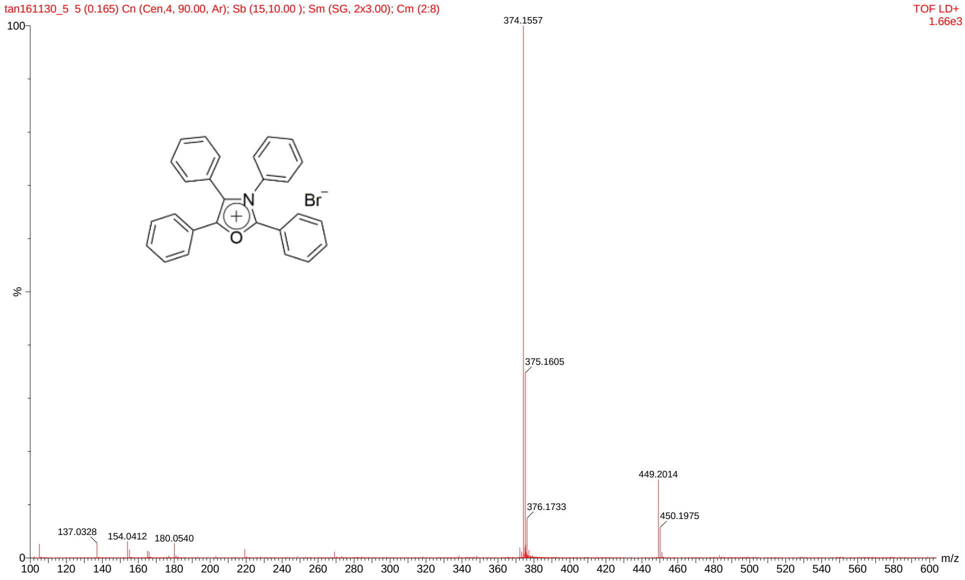

Supplementary Figure 7 HRMS spectrum of TPO-Br.

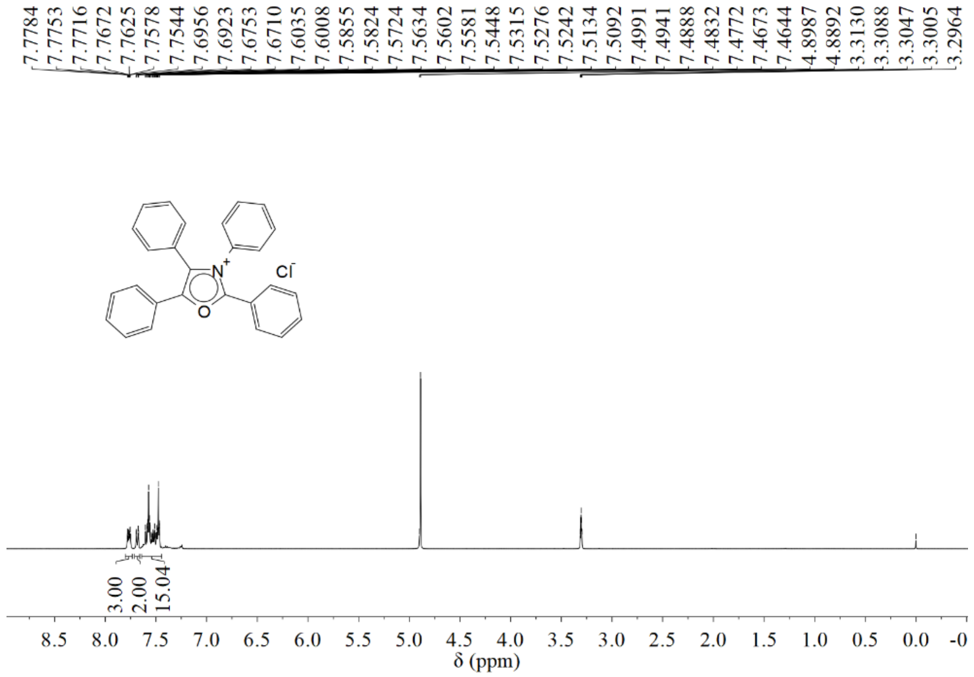

Supplementary Figure 8 ^{1}H NMR spectrum of TPO-Cl in CD_3OD.

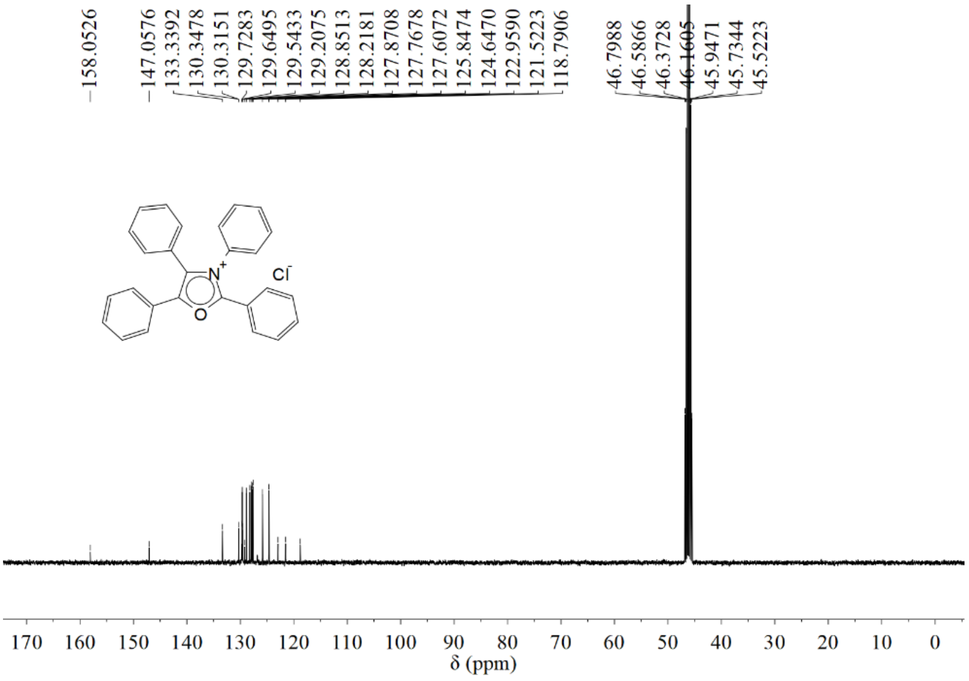

Supplementary Figure 9 ^{13}C NMR spectrum of TPO-Cl in CD_3OD.

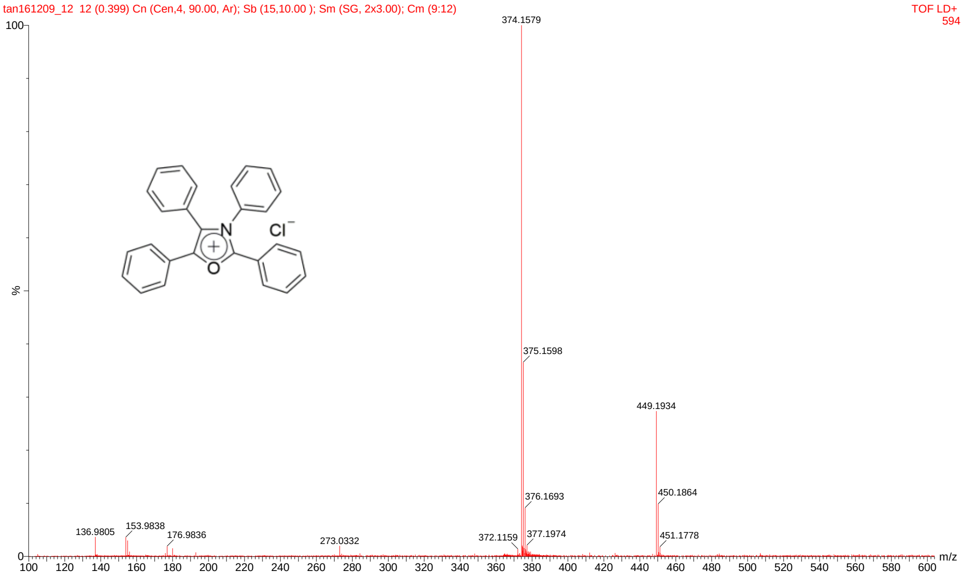

Supplementary Figure 10 HRMS spectrum of TPO-Cl.

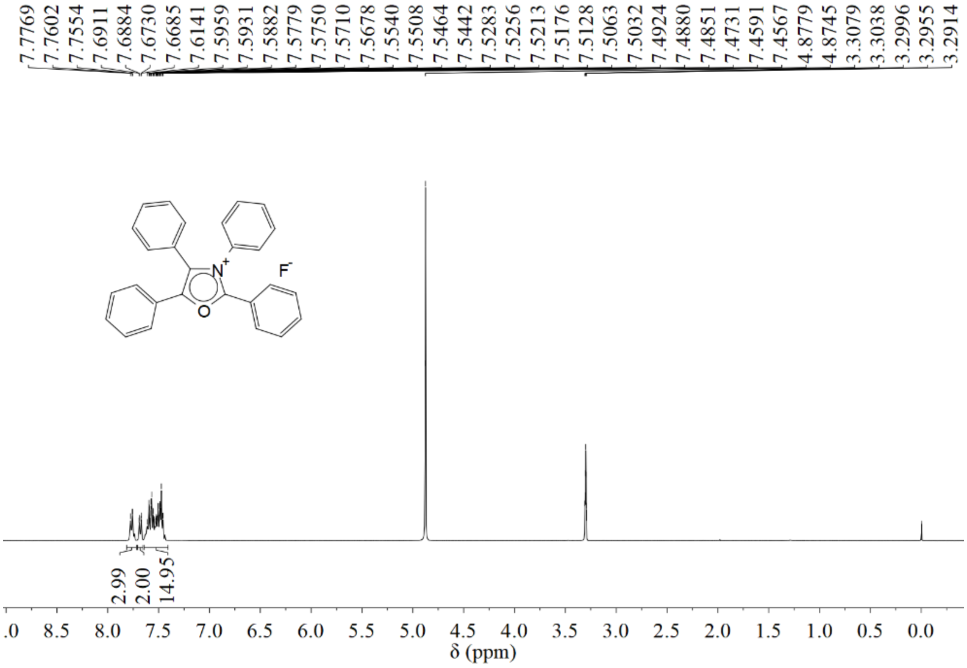

Supplementary Figure 11 ^{1}H NMR spectrum of TPO-F in CD_3OD.

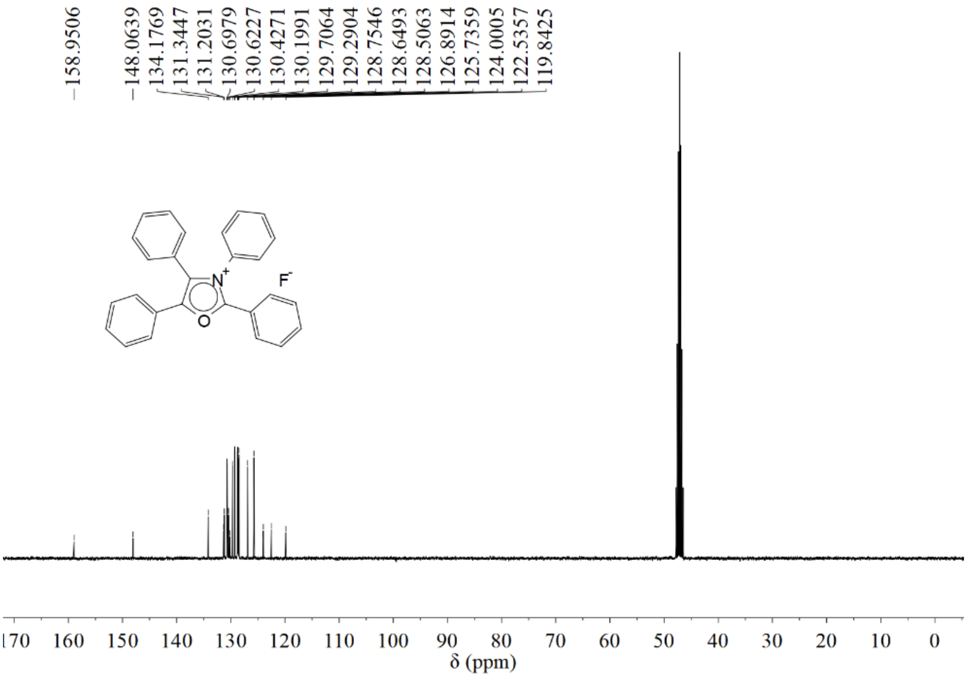

Supplementary Figure 12 ^{13}C NMR spectrum of TPO-F in CD_3OD.

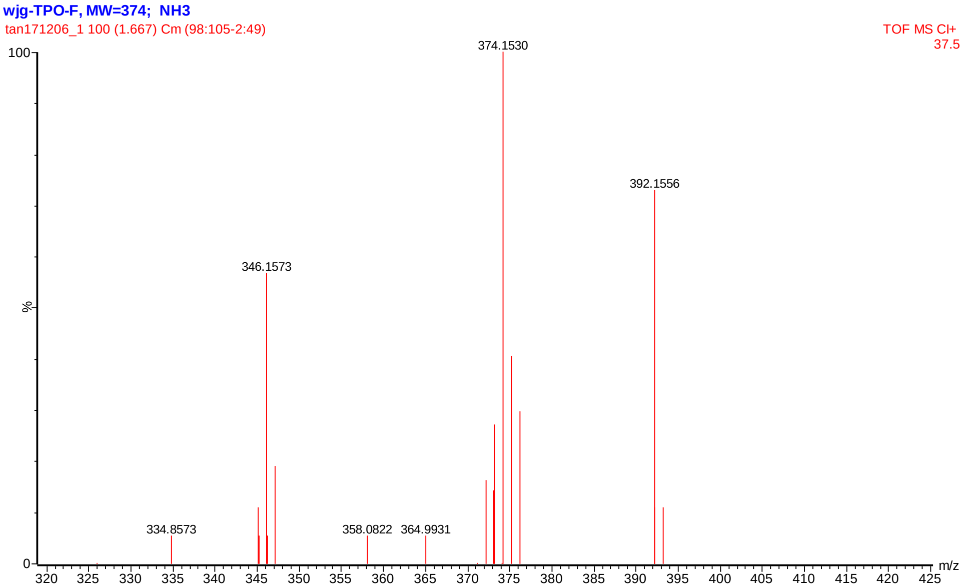

Supplementary Figure 13 HRMS spectrum of TPO-F.

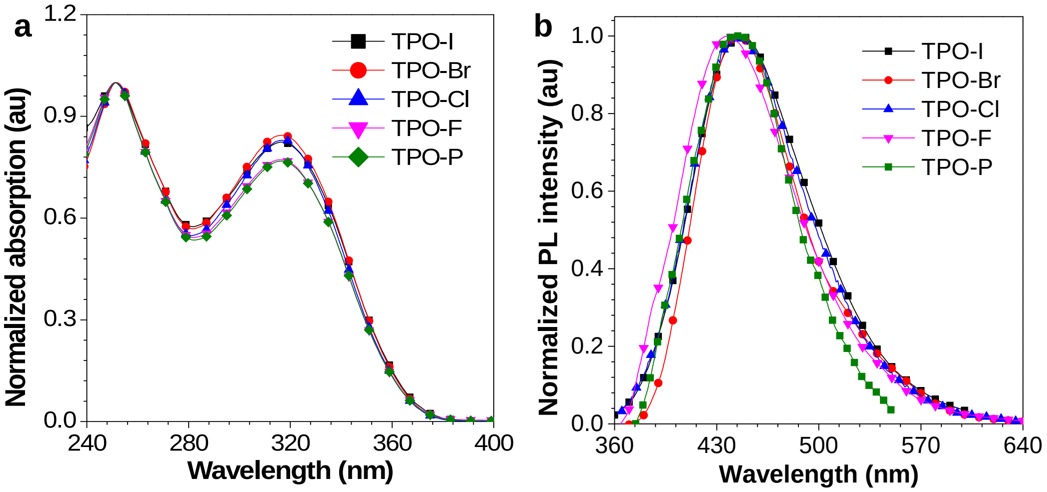

Supplementary Figure 14 a UV-vis and **b** Photoluminescence (PL) spectra of TPO-I, TPO-Br, TPO-Cl, TPO-F and TPO-P (10 μM) in EtOH solution.

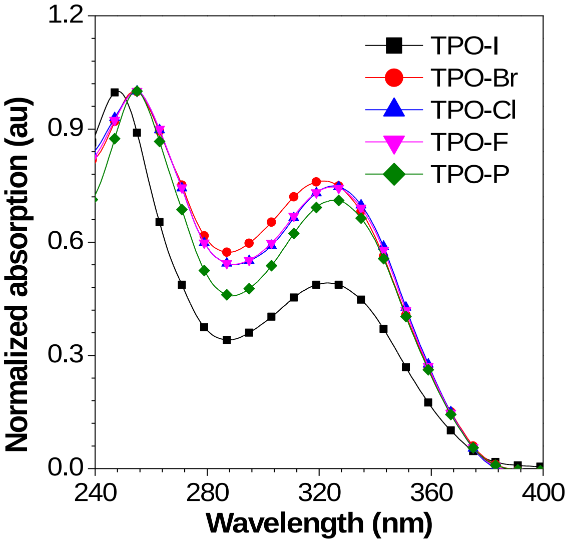

Supplementary Figure 15 UV-vis spectra of TPO-I, TPO-Br, TPO-Cl, TPO-F and TPO-P (10 μM) in DCM solution.

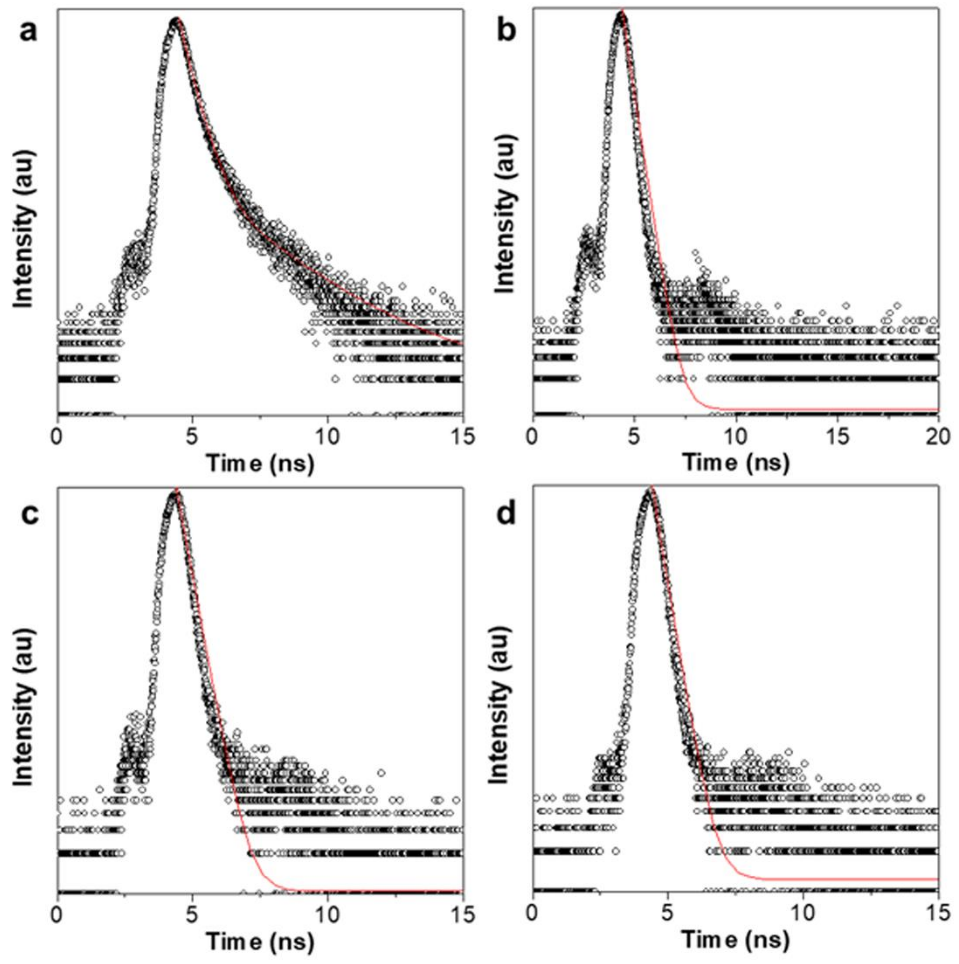

Supplementary Figure 16 Time-resolved PL decay of **a** TPO-I, **b** TPO-Br, **c** TPO-Cl, and **d** TPO-F at maximum emission wavelength in EtOH solution.

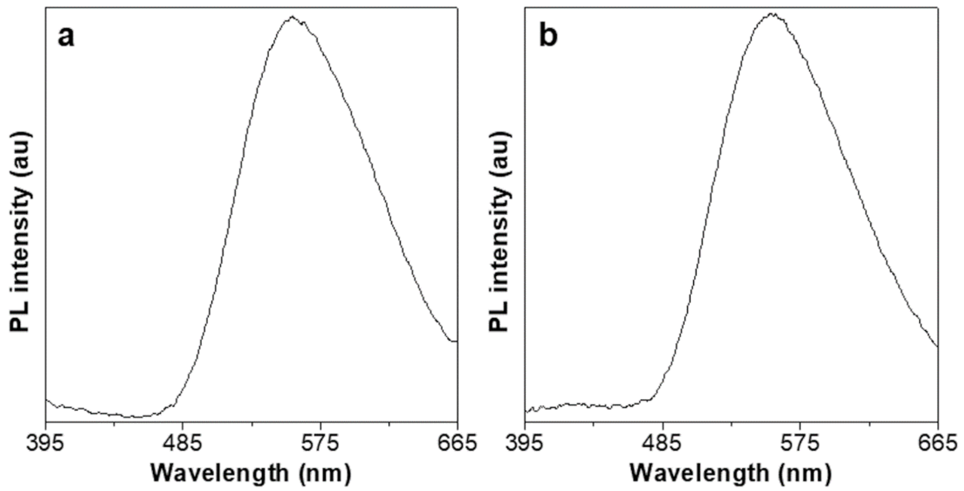

Supplementary Figure 17 The delayed (50 μs) PL spectra of powder of **a** TPO-I and **b** TPO-Br.

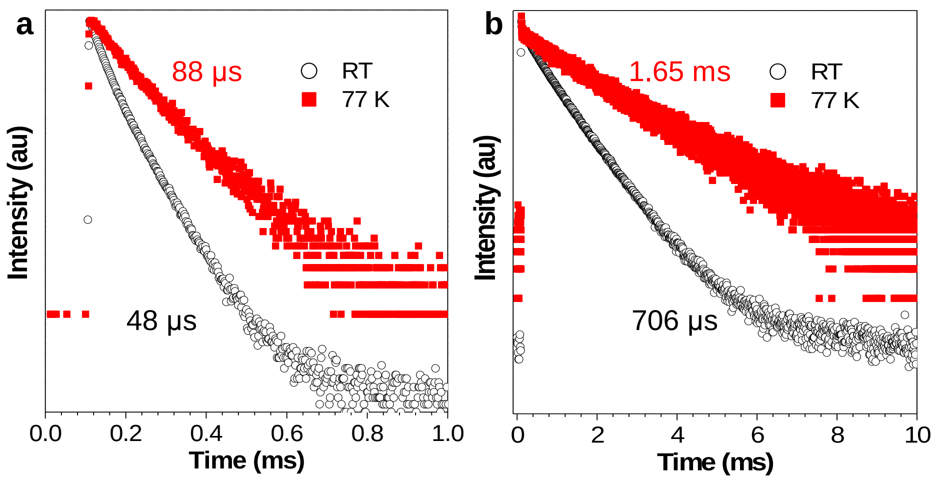

Supplementary Figure 18 Time-resolved PL decay of powders of **a** TPO-I at 559 nm and **b** TPO-Br at 549 nm under different temperature.

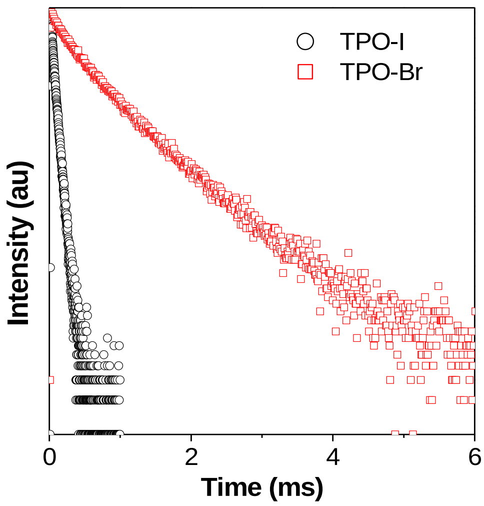

Supplementary Figure 19 Time-resolved PL decay of TPO-I (@ 559 nm) and TPO-Br (@ 549 nm) in powder in vacuum.

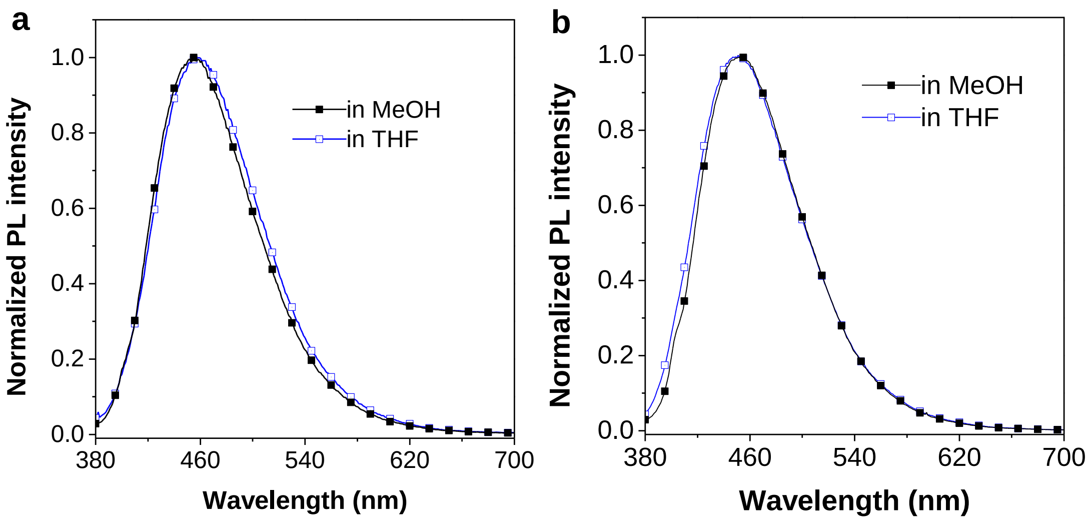

Supplementary Figure 20 PL spectra of **a** TPO-I and **b** TPO-Br in different solvents (THF and MeOH) with different dielectric constants at room temperature. The dielectric constants of THF and MeOH are 7.58 and 32.7, respectively. Their emission peaks are at about 450 nm.

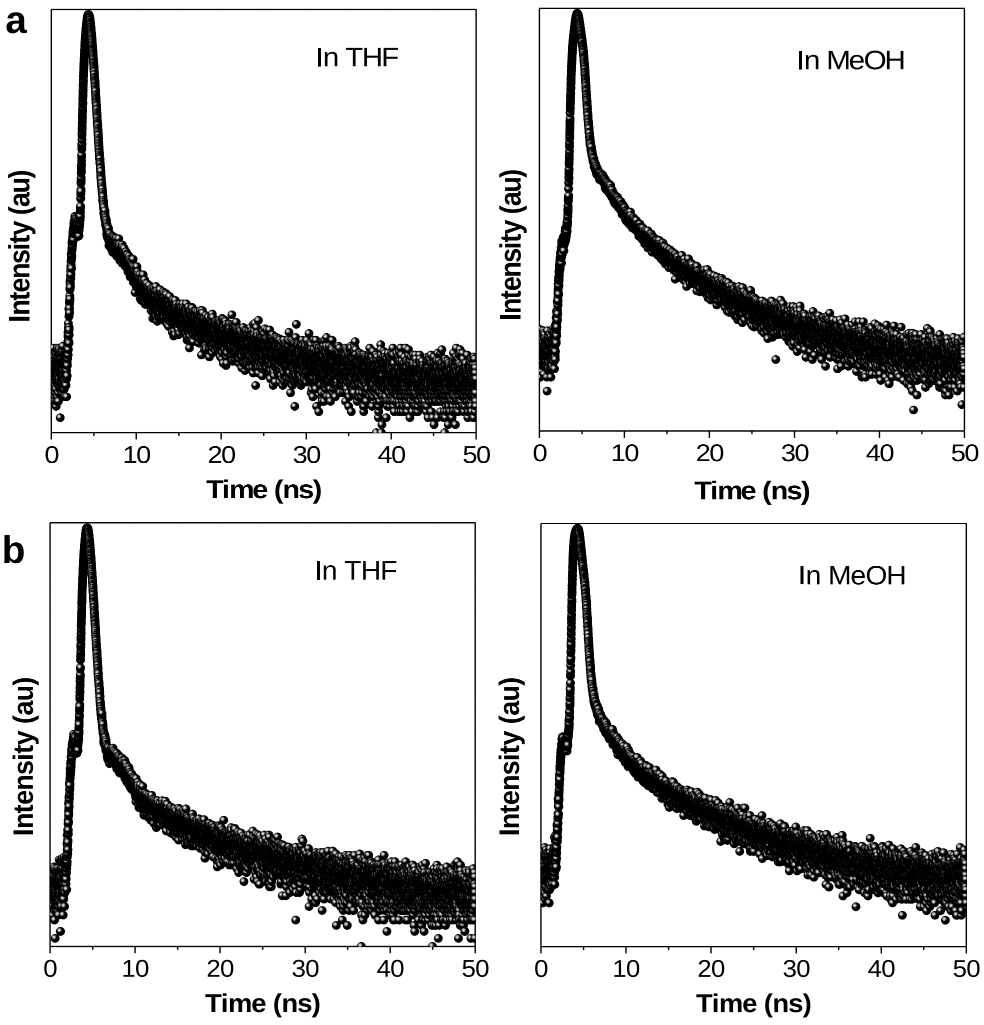

Supplementary Figure 21 Time-resolved PL decay of **a** TPO-I (@ 450 nm) and **b** TPO-Br (@ 450 nm) in different solvents at the room temperature (λ_{ex} = 375 nm) under air.

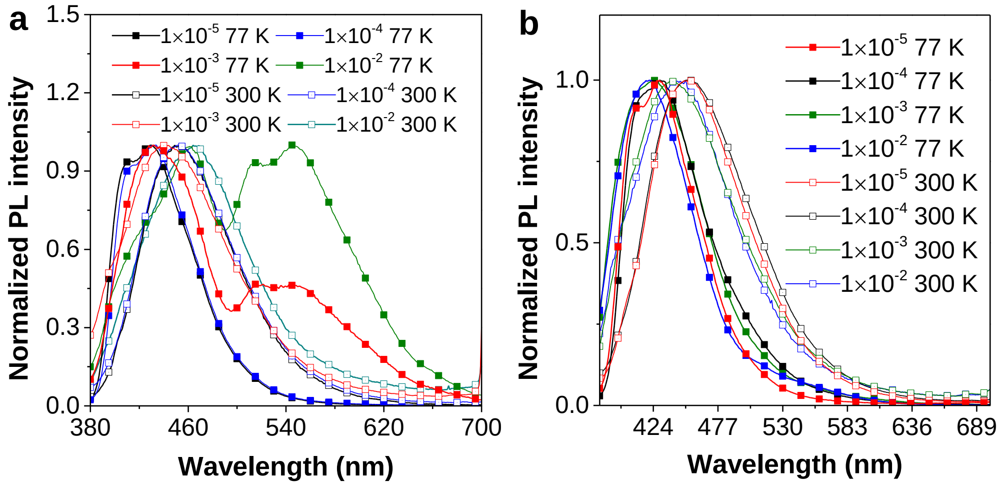

Supplementary Figure 22 PL spectra of **a** TPO-I and **b** TPO-Br with different concentrations at 300 K and 77 K.

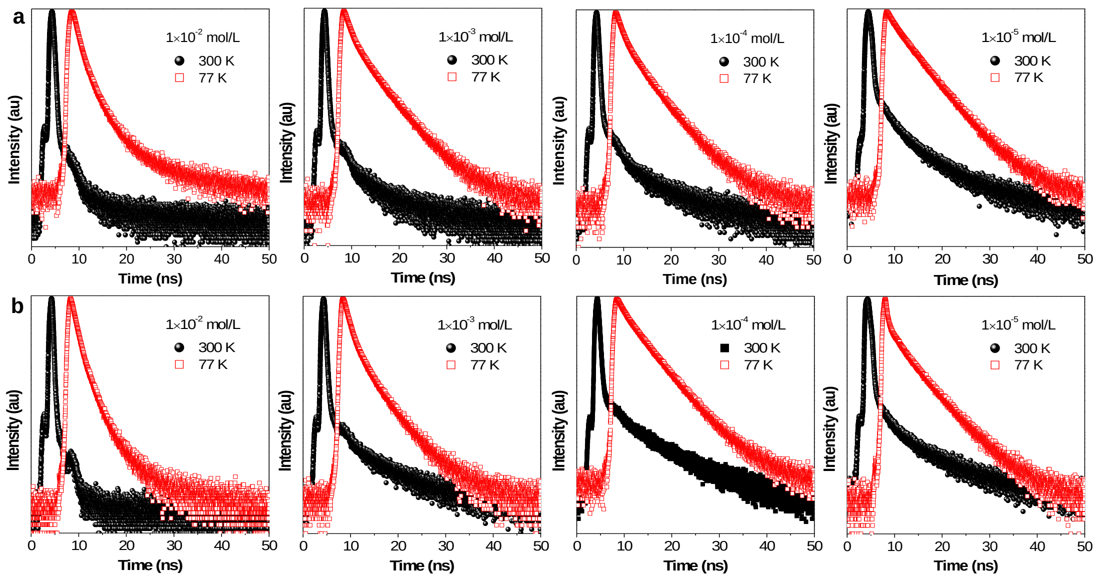

Supplementary Figure 23 Time-resolved PL decay of **a** TPO-I (@ 447 nm) and **b** TPO-Br (@ 444 nm) with the different concentrations in MeOH at 300 K and 77 K (λ_{ex} = 375 nm) under air.

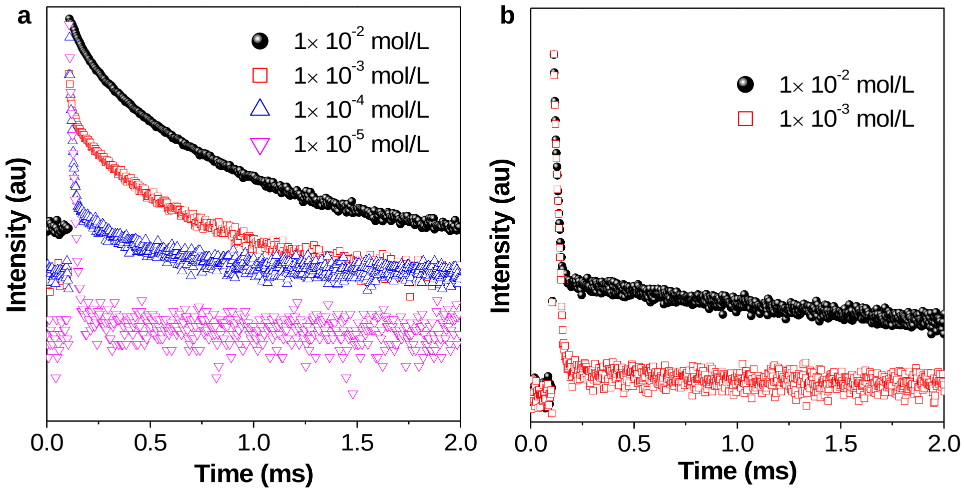

Supplementary Figure 24 Time-resolved PL decay of **a** TPO-I (@ 559 nm) and **b** TPO-Br (@ 549 nm) with the different concentrations in MeOH at 77 K (λ_{ex} = 350 nm) under air.

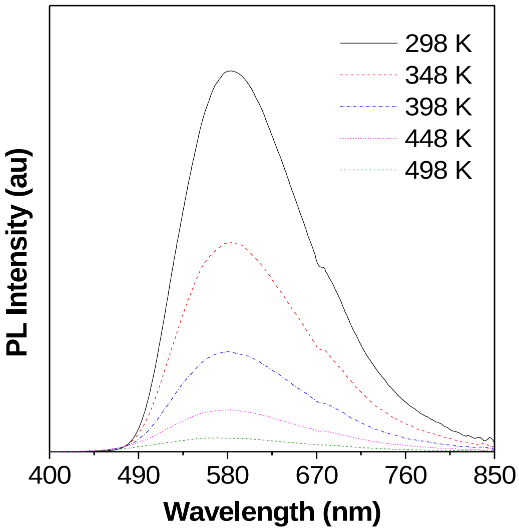

Supplementary Figure 25 PL spectra of TPO-I powder at different temperature.

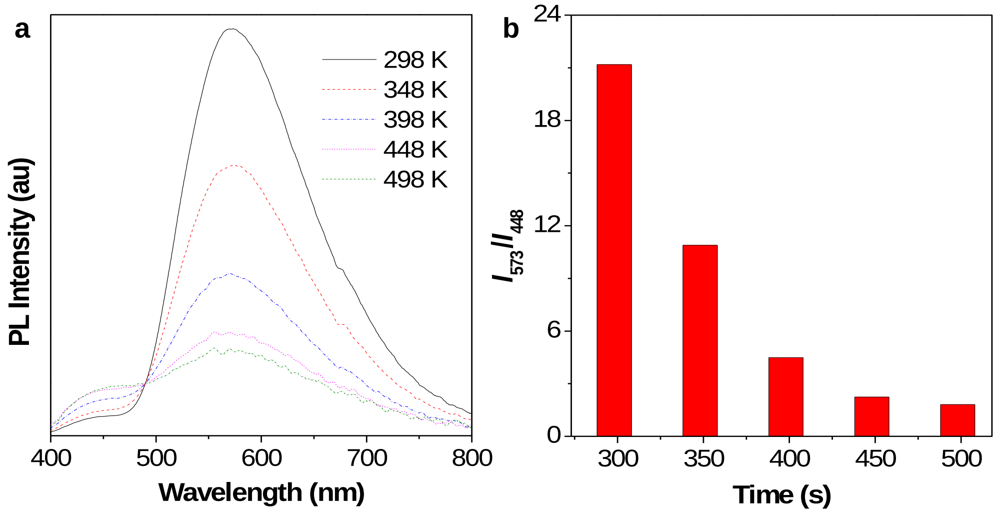

Supplementary Figure 26 a PL spectra and **b** phosphorescence (@ 573 nm)/fluorescence (@ 448 nm) intensity ratio of TPO-Br powder at different temperature.

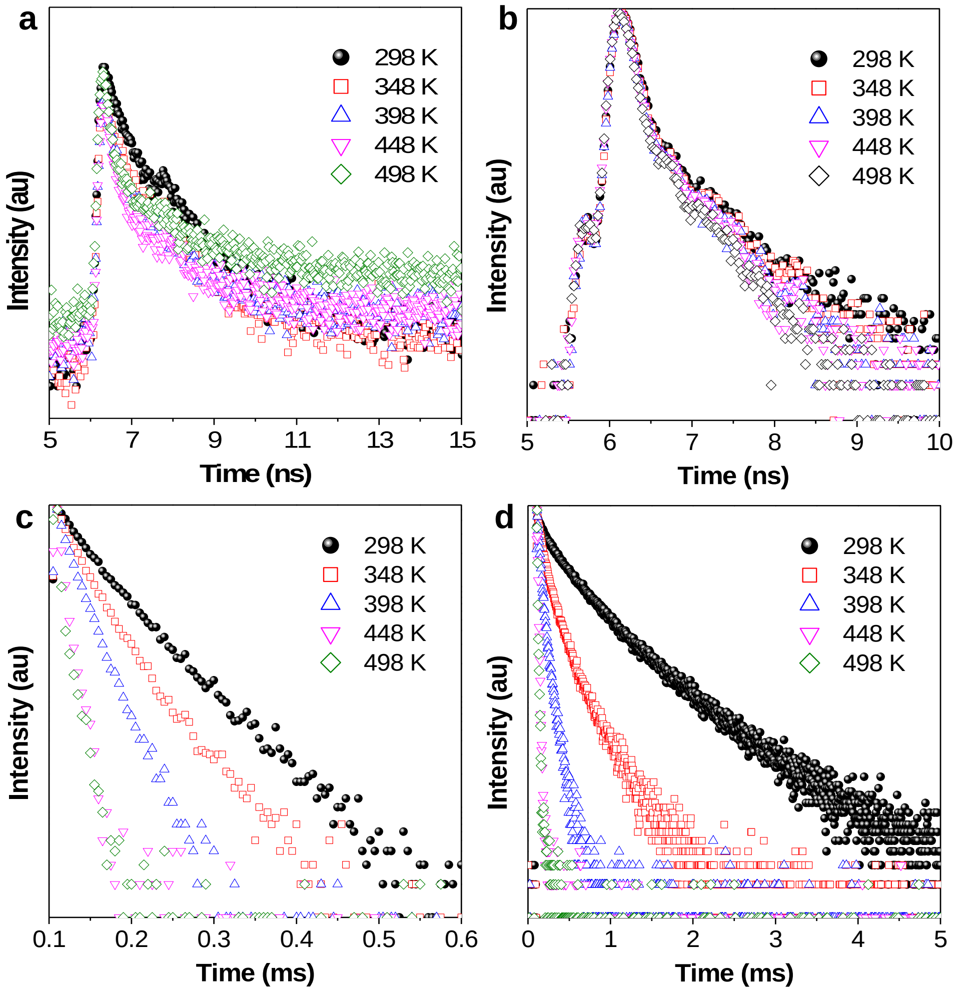

Supplementary Figure 27 Time-resolved PL decay of powders of TPO-I **a** @ 450 nm, **c** @ 559 nm and TPO-Br **b** @ 434 nm, **d** @ 549 nm at different temperature under air.

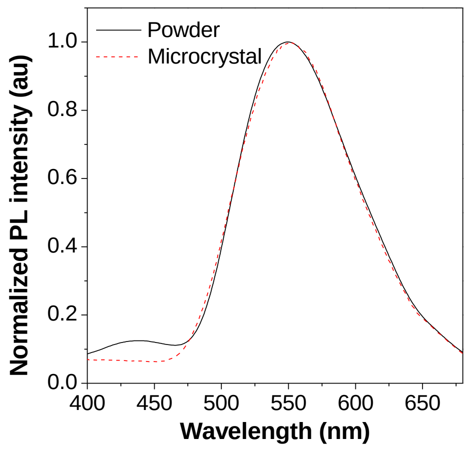

Supplementary Figure 28 PL spectra of powder and microcrystal of TPO-Br.

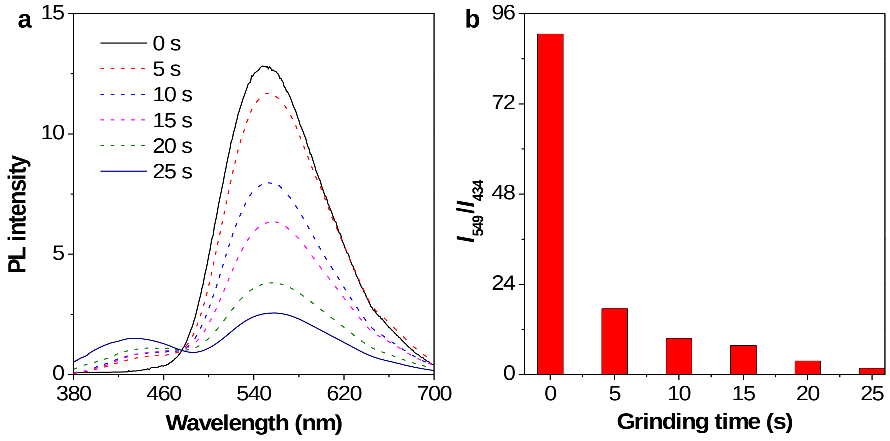

Supplementary Figure 29 a PL spectra and **b** the intensity ratio between phosphorescence (@ 549 nm) and fluorescence (@ 434 nm) for TPO-Br microcrystals under grinding for different time.

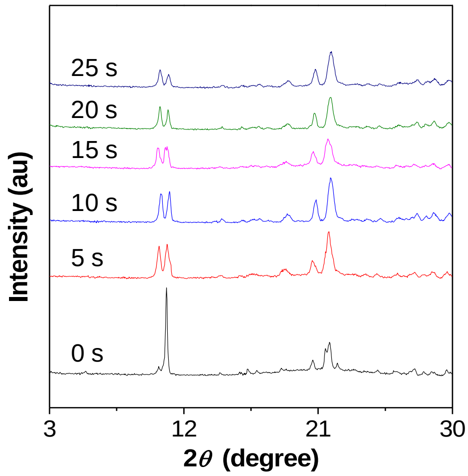

Supplementary Figure 30 XRD patterns of TPO-Br microcrystals under grinding for different time.

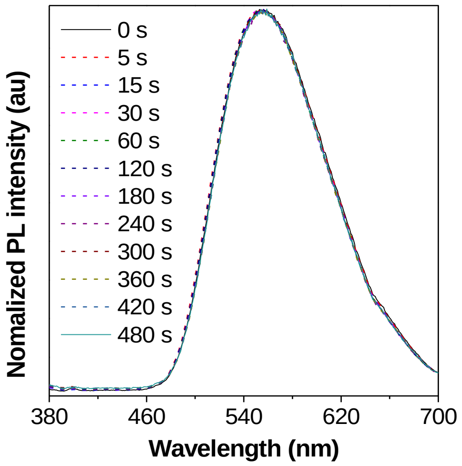

Supplementary Figure 31 PL spectra of TPO-I powder under grinding for different time.

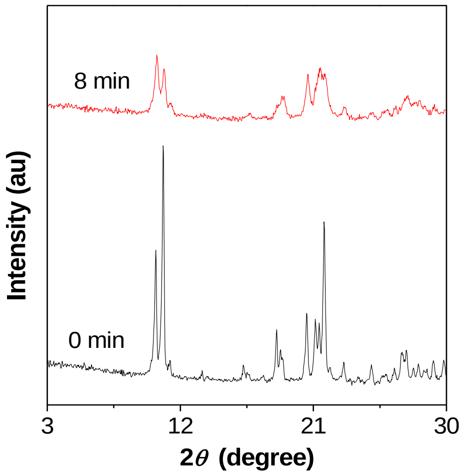

Supplementary Figure 32 XRD patterns of TPO-I powder before and after grinding for 8 min.

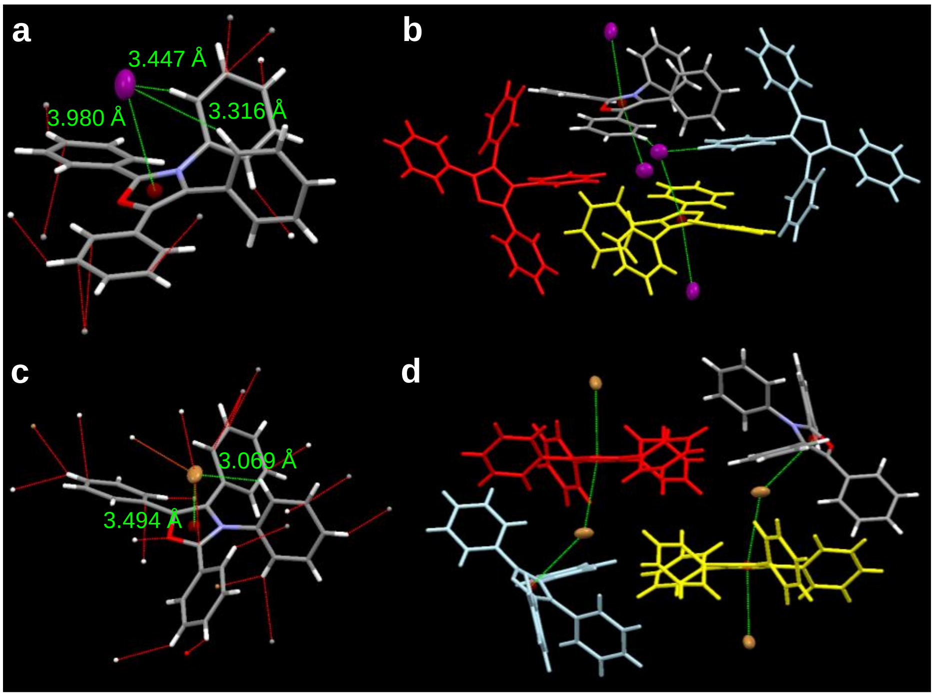

Supplementary Figure 33 a and **c** Short contacts, hydrogen bonding, anion-π^+ interactions and **b** and **d** intermolecular stacking of TPO-I (top) and TPO-Br (bottom), respectively.

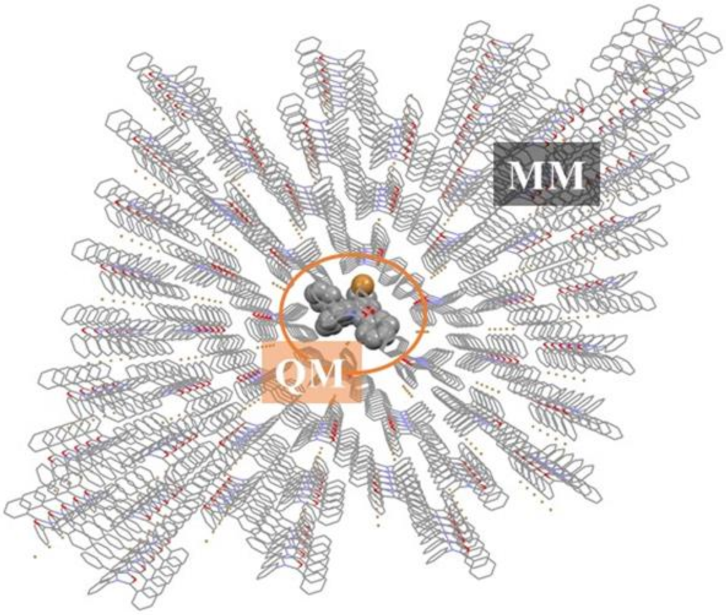

Supplementary Figure 34 QM/MM model taking TPO-Br as an example: one central QM molecule for the higher layer and the remain MM molecules for the lower layer.

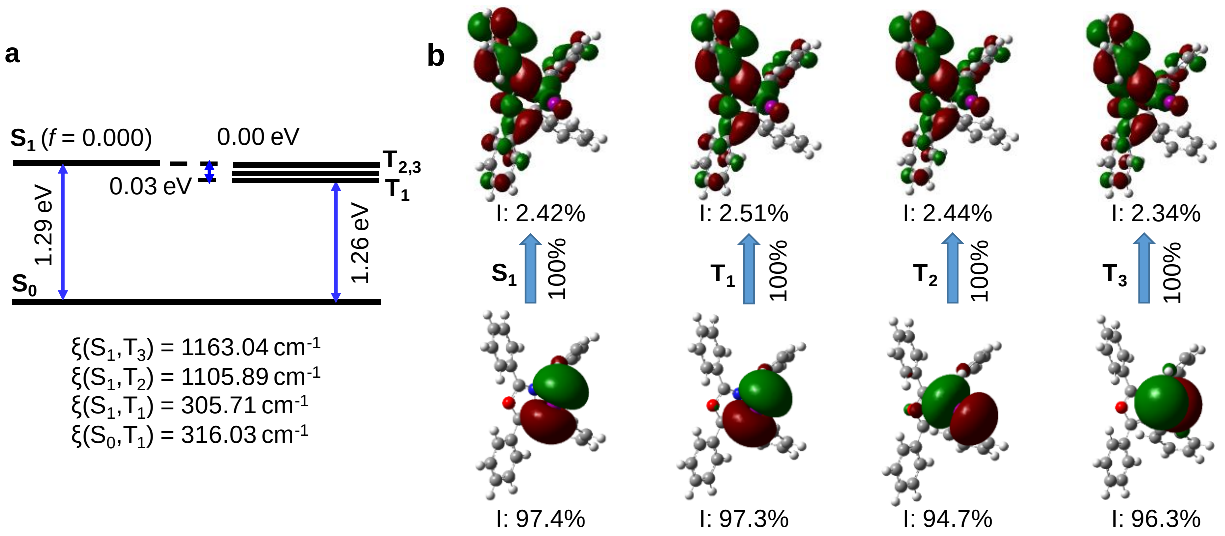

Supplementary Figure 35 The calculated energy level diagram, spin-orbit couplings (ξ) between singlet and triplet states, and the oscillator strengths (*f*) of the S_1 state of **a** TPO-I in crystal based on the optimized ground-state geometries using ONIOM method. The natural transition orbitals (NTOs) (hole ones at the bottom and electron ones on the top) and the corresponding proportions for **b** TPO-I.

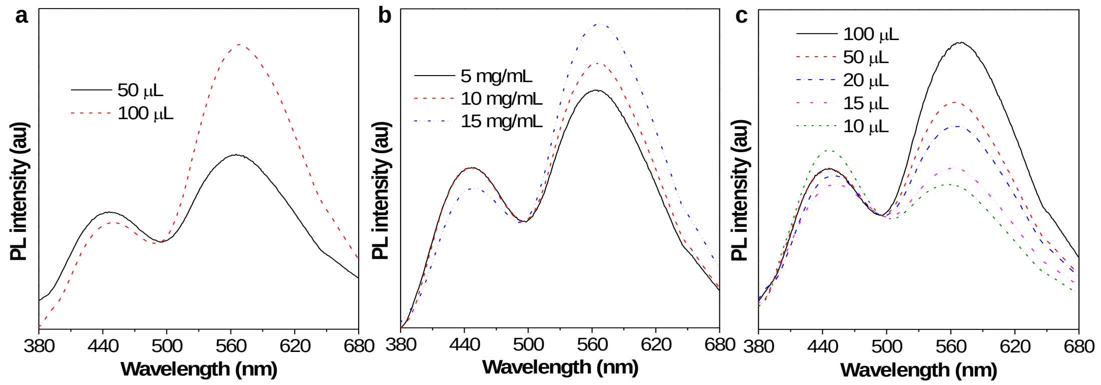

Supplementary Figure 36 PL spectra of TPO-Br films which were made to take **a** 10 mg/mL TPO-Br in MeOH solution with different volume on quartz plate, **b** 50 μL TPO-Br in MeOH solution with different concentration on quartz plate and **c** 5 mg/mL TPO-Br in MeOH solution with different volume on quartz plate.

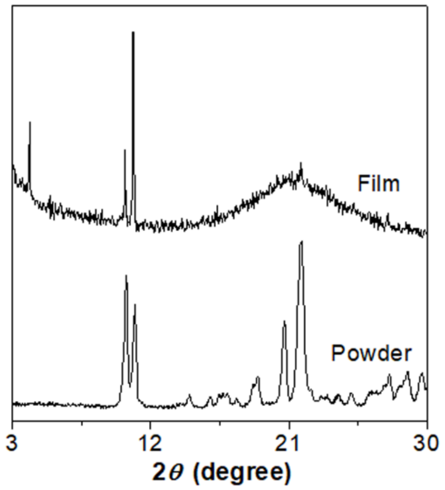

Supplementary Figure 37 XRD patterns of film and powder of TPO-Br.

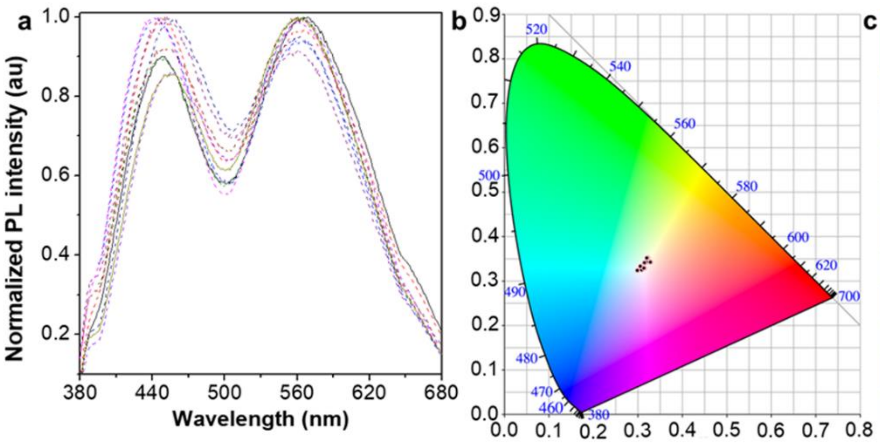

c

Time (day)	CIE x	CIE y
3	0.33	0.34
6	0.32	0.33
9	0.31	0.32
12	0.31	0.33
15	0.32	0.34
18	0.31	0.33
21	0.32	0.35
24	0.30	0.32
27	0.31	0.34
30	0.32	0.35

Supplementary Figure 38 a PL spectra and **b** CIE 1931 coordinates and **c** CIE value of films of TPO-Br were monitored at room temperature (22 ºC) during one month. Excitation wavelength: 347 nm.

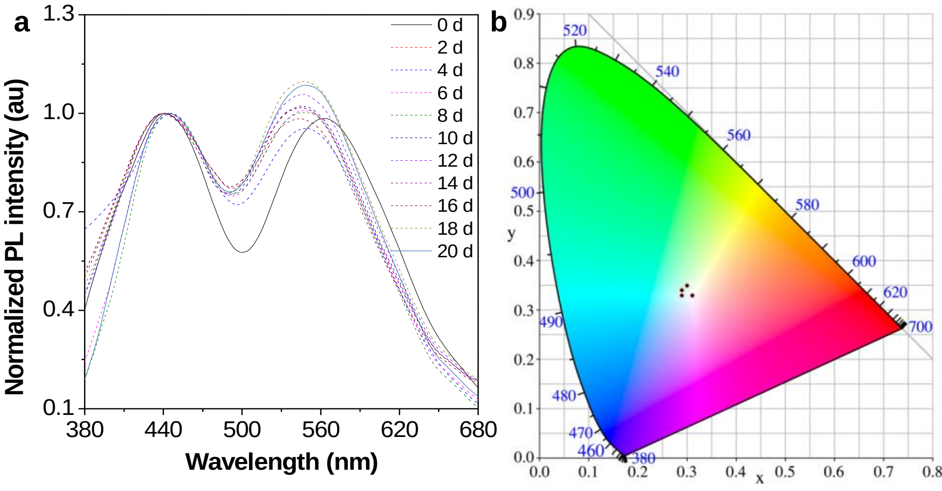

c

Time (day)	CIE x	CIE y
0	0.31	0.33
2	0.29	0.34
4	0.29	0.33
6	0.29	0.34
8	0.29	0.34
10	0.29	0.34
12	0.29	0.34
14	0.29	0.34
16	0.29	0.33
18	0.30	0.35
20	0.30	0.35

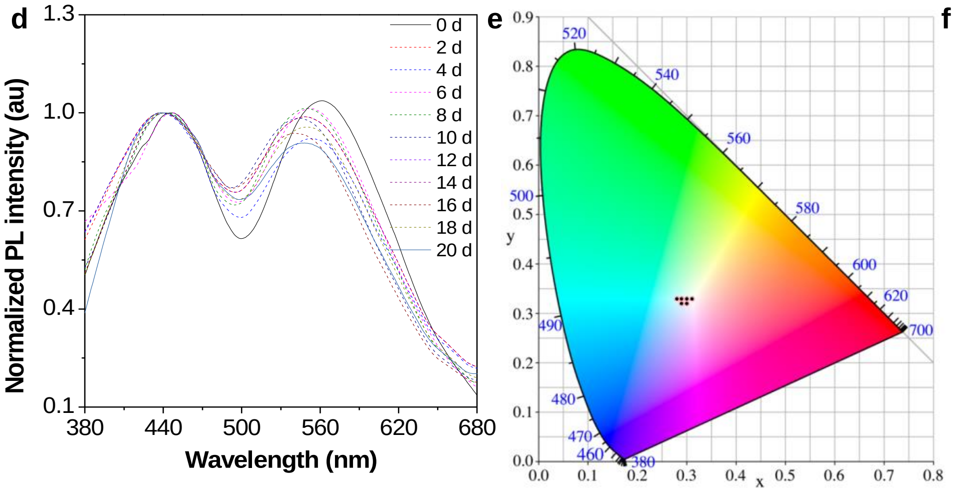

f

Time (day)	CIE x	CIE y
0	0.31	0.33
2	0.30	0.33
4	0.30	0.32
6	0.30	0.33
8	0.30	0.33
10	0.29	0.33
12	0.30	0.33
14	0.30	0.33
16	0.28	0.33
18	0.29	0.33
20	0.29	0.32

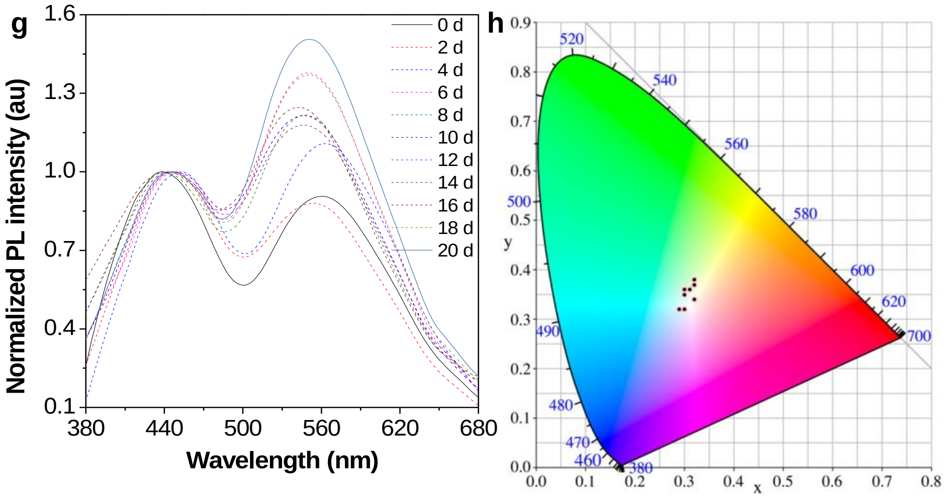

i

Time (day)	CIE x	CIE y
0	0.30	0.32
2	0.29	0.32
4	0.32	0.34
6	0.32	0.37
8	0.31	0.36
10	0.30	0.36
12	0.30	0.36
14	0.30	0.35
16	0.30	0.36
18	0.32	0.37
20	0.32	0.38

Supplementary Figure 39 PL spectra (**a**, **d** and **g**), CIE 1931 coordinates (**b**, **e** and **h**) and CIE value (**c**, **f** and **i**) of films of TPO-Br were monitored at (**a, b** and **c**) -20 °C, (**d**, **e** and **f**) 40 °C and (**g**, **h** and **i**) 70 °C during 20 days.

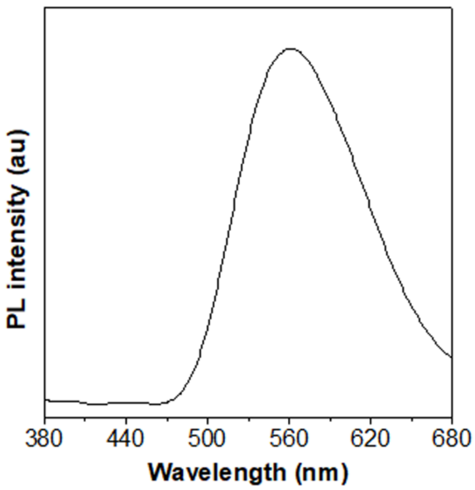

Supplementary Figure 40 PL spectra of TPO-I film.

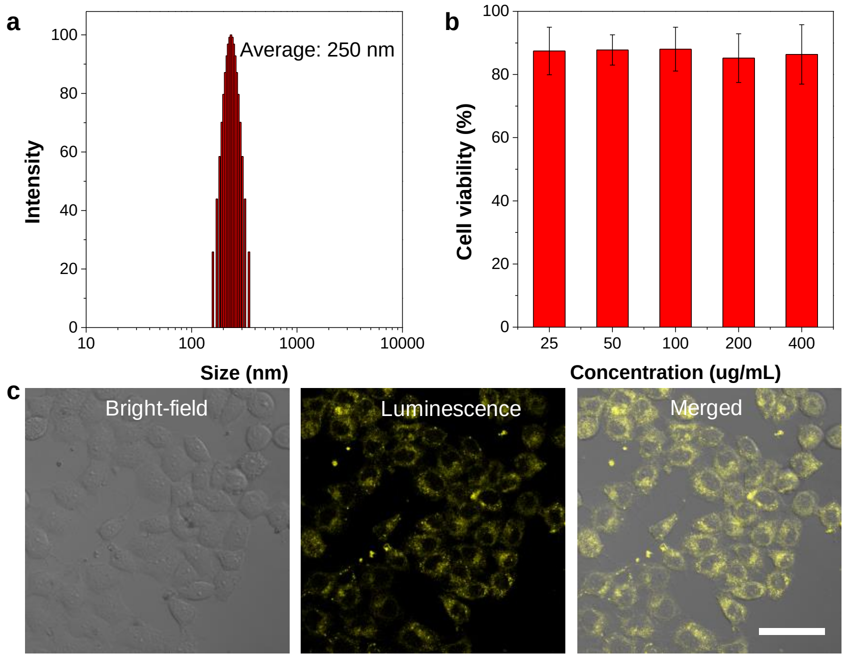

Supplementary Figure 41 **a** DLS analysis of DSPE-PEG-encapsulated TPO-Br NPs. **b** Cell viabilities of HeLa cells in the presence of different concentrations of TPO-Br NPs. Error bars are defined as s.d. **c** Bright-field, luminescent and merged images of HeLa cells stained with TPO-Br NPs (1 mg/mL) based on laser scanning confocal microscope (LSCM). Excitation wavelength: 405 nm, emission was acquired in the range from 500 to 720 nm. Scale bar: 50 μm.

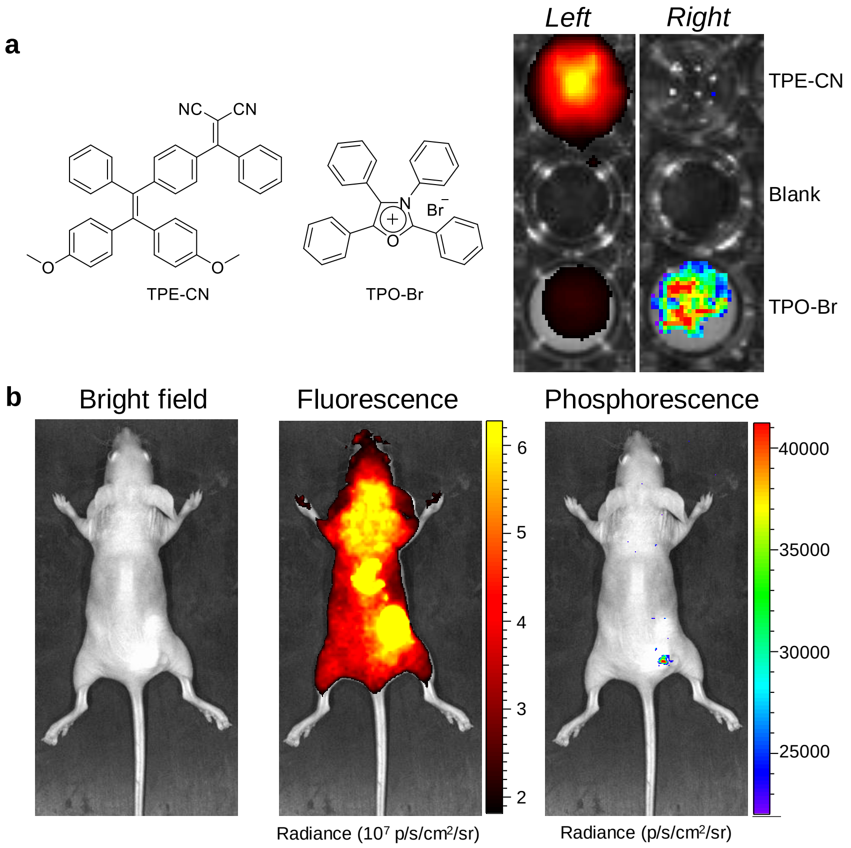

Supplementary Figure 42 *In vivo* phosphorescent imaging. **a** Luminescent photos of the aqueous solutions of DSPE-PEG-encapsulated TPE-CN NPs and TPO-Br NPs (*Left*: fluorescence; *Right*: phosphorescence). Blank is the DSPE-PEG solution. **b** Luminescent imaging in living mice after subcutaneous injection of TPE-CN NPs (fluorescence) and TPO-Br NPs (Phosphorescence).

Supplementary Table 1 Fluorescence lifetime data of AIEgens in EtOH at room temperature (298 K; $\lambda_{ex} = 375$ nm) under air.

	TPO-I	TPO-Br	TPO-Cl	TPO-F	TPO-P
λ_{em} (nm)	447	444	443	438	444
τ_1 (ns) / A_1 (%)	0.46 / 96.43	0.40 / 100	0.39 / 100	0.36 / 100	0.86 / 100
τ_2 (ns) / A_2 (%)	2.61 / 3.57	/	/	/	/
τ (ns) [a]	0.83	0.40	0.39	0.36	0.86

[a] τ = average fluorescence lifetime at 434 nm calculated by $\tau = \Sigma A_i \tau_i^2 / \Sigma A_i \tau_i$, where A_i is the pre-exponential factor for lifetime τ_i.

Supplementary Table 2 Fluorescence lifetime data of AIEgens in powder at room temperature (298 K; λ_{ex} = 375 nm) under air and in vacuum.

	TPO-Br		TPO-Cl		TPO-F		TPO-P	
	under air	in vacuum	under air	in vacuum	under air	in vacuum	under air	in vacuum
λ_{em} (nm)	434	434	435	435	420	420	422	422
τ_1 (ns) / A_1 (%)	0.75 / 91.75	0.05 / 92.63	0.83 / 82.62	0.95 / 86.04	0.80 / 100	0.74 / 83.00	1.02 / 100	0.49 / 81.51
τ_2 (ns) / A_2 (%)	5.30 / 8.25	2.14 / 7.37	2.69 / 17.38	2.81 / 3.96	/	2.37 / 17.00	/	1.28 / 18.49
τ (ns) [a]	2.52	1.64	1.60	1.55	0.80	1.39	1.02	0.78

[a] τ = average fluorescence lifetime at 434 nm calculated by $\tau = \Sigma A_i\tau_i^2/\Sigma A_i\tau_i$, where A_i is the pre-exponential factor for lifetime τ_i.

Supplementary Table 3 Phosphorescence lifetime data of AIEgens in powder at 77 K under air (λ_{ex} = 350 nm).

	TPO-I	TPO-Br
λ_{em} (nm)	559	549
τ_1 (μs) / A_1 (%)	88.16 / 100	1646.75 / 100
τ (μs) [a]	88.16	1646.75

[a] τ = average phosphorescence lifetime calculated by $\tau = \Sigma A_i\tau_i^2/\Sigma A_i\tau_i$, where A_i is the pre-exponential factor for lifetime τ_i.

Supplementary Table 4 Phosphorescence lifetime data of AIEgens in powder at room temperature (298 K; λ_{ex} = 350 nm) under air and in vacuum.

	TPO-I		TPO-Br	
	under air	in vacuum	under air	in vacuum
λ_{em} (nm)	559	559	549	549
τ_1 (μs) / A_1 (%)	66.09 / 41.30	9.42 / 2.24	204.00 / 20.14	92.31 / 1.94
τ_2 (μs) / A_2 (%)	27.66 / 58.70	54.65 / 77.76	743.12 / 76.25	388.80 / 4.74
τ_3 (μs) / A_3 (%)	/	/	5.79 / 3.61	858.40 / 63.32
τ (μs) [a]	48.74	52.52	706.42	763.16

[a] τ = average phosphorescence lifetime calculated by $\tau = \Sigma A_i\tau_i^2/\Sigma A_i\tau_i$, where A_i is the pre-exponential factor for lifetime τ_i.

Supplementary Table 5 Fluorescence lifetime data of TPO-I in different solvents with different dielectric constants at room temperature (λ_{ex} = 375 nm, λ_{em} = 450 nm) under air.

	THF [b]	MeOH [b]
τ_1 (ns) / (%)	4.37 / 3.44	4.47 / 8.96
τ_2 (ns) / (%)	0.31 / 96.56	0.28 / 91.04
τ (μs) [a]	1.67	2.84

[a] τ = average fluorescence lifetime calculated by $\tau = \Sigma A_i\tau_i^2/\Sigma A_i\tau_i$, where A_i is the pre-exponential factor for lifetime τ_i.
[b]The dielectric constants of THF and MeOH are 7.58 and 32.7, respectively.

Supplementary Table 6 Fluorescence lifetime data of TPO-Br in different solvents with different dielectric constants at room temperature (λ_{ex} = 375 nm, λ_{em} = 450 nm) under air.

	THF	MeOH
τ_1 (ns) / (%)	4.98 / 0.01	0.37 / 94.65
τ_2 (ns) / (%)	0.30 / 99.99	6.17 / 5.35
τ (ns) [a]	0.30	3.18

[a] τ = average fluorescence lifetime calculated by $\tau = \Sigma A_i\tau_i^2/\Sigma A_i\tau_i$, where A_i is the pre-exponential factor for lifetime τ_i.
[b]The dielectric constants of THF and MeOH are 7.58 and 32.7, respectively.

Supplementary Table 7 Fluorescence lifetime data of TPO-I with different concentrations in MeOH at 300 K and 77 K (λ_{ex} = 375 nm, λ_{em} = 447 nm) under air.

	1×10^{-2} (mol/L)		1×10^{-3} (mol/L)		1×10^{-4} (mol/L)		1×10^{-5} (mol/L)	
	300 K	77 K	300 K	77 K	300 K	77 K	300 K	77 K
τ_1 (ns) /	0.23 /	3.91 /	3.99 /	4.76 /	0.29 /	4.70 /	4.47 /	1.71 /
(%)	96.46	18.86	1.85	48.79	98.50	45.19	8.96	21.56
τ_2 (ns) /	3.25 /	1.16 /	0.26 /	1.22/	4.86 /	1.18 /	0.28 /	5.01 /
(%)	3.54	81.14	98.15	51.21	1.50	54.81	91.04	78.44
τ (ns) [a]	1.26	2.37	1.10	4.01	1.22	3.88	2.84	4.72

[a] τ = average fluorescence lifetime calculated by $\tau = \Sigma A_i\tau_i^2/\Sigma A_i\tau_i$, where A_i is the pre-exponential factor for lifetime τ_i.

Supplementary Table 8 Fluorescence lifetime data of TPO-Br with different concentrations in MeOH at 300 K and 77 K (λ_{ex} = 375 nm, λ_{em} = 444 nm) under air.

	1×10^{-2} (mol/L)		1×10^{-3} (mol/L)		1×10^{-4} (mol/L)		1×10^{-5} (mol/L)	
	300 K	77 K	300 K	77 K	300 K	77 K	300 K	77 K
τ_1 (ns) /	0.2 /	3.63 /	5.48 /	4.37 /	7.00 /	4.99 /	0.37 /	5.01 /
(%)	97.99	12.42	2.08	26.89	2.06	75.88	94.65	31.43
τ_2 (ns) /	2.34/	1.15 /	0.27 /	1.16/	0.32 /	1.44 /	6.17 /	0.66 /
(%)	2.01	87.58	97.91	73.11	97.94	24.12	5.35	68.57
τ (ns) [a]	0.61	1.92	1.84	3.02	2.43	4.69	3.18	4.03

[a] τ = average fluorescence lifetime calculated by $\tau = \Sigma A_i\tau_i^2/\Sigma A_i\tau_i$, where A_i is the pre-exponential factor for lifetime τ_i.

Supplementary Table 9 Phosphorescence lifetime data of TPO-I with different concentrations in MeOH at 300 K and 77 K (λ_{ex} = 350 nm, λ_{em} = 559 nm) under air.

	1×10^{-2} (mol/L)		1×10^{-3} (mol/L)		1×10^{-4} (mol/L)		1×10^{-5} (mol/L)	
	300 K	77 K	300 K	77 K	300 K	77 K	300 K	77 K
τ_1 (μs) / (%)	ND [a]	365 / 15.42	ND	256 / 12.07	ND	100 / 2.19	ND	7.5 / 100
τ_2 (μs) / (%)	ND	125 / 43.67	ND	57 / 16.11	ND	7 / 96.52	ND	/
τ_3 (μs) / (%)	ND	35 / 40.91	ND	6 / 71.82	ND	371 / 1.29	ND	/
τ (μs) [b]	ND	222	ND	199	ND	149	ND	7.5

[a] No detectable. [b] τ = average phosphorescence lifetime calculated by $\tau = \Sigma A_i\tau_i^2/\Sigma A_i\tau_i$, where A_i is the pre-exponential factor for lifetime τ_i.

Supplementary Table 10 Phosphorescence lifetime data of TPO-Br with different concentrations in MeOH at 300 K and 77 K (λ_{ex} = 350 nm, λ_{em} = 549 nm) under air.

	1×10^{-2} (mol/L)		1×10^{-3} (mol/L)		1×10^{-4} (mol/L)		1×10^{-5} (mol/L)	
	300 K	77 K	300 K	77 K	300 K	77 K	300 K	77 K
τ_1 (μs) / (%)	ND [a]	5.6 / 100	ND	ND	ND	ND	ND	ND
τ_2 (μs) / (%)	ND	/	ND	ND	ND	ND	ND	ND
τ (μs) [b]	ND	5.6	ND	ND	ND	ND	ND	ND

[a] No detectable. [b] τ = average phosphorescence lifetime calculated by $\tau = \Sigma A_i\tau_i^2/\Sigma A_i\tau_i$, where A_i is the pre-exponential factor for lifetime τ_i.

Supplementary Table 11 Fluorescence lifetime data of TPO-I powder at different temperature under air (λ_{ex} = 375 nm, λ_{em} = 450 nm).

	298 K	348 K	398 K	448 K	498 K
τ_1 (ns) / (%)	0.14 / 20.86	0.11 / 20.59	0.09 / 25.22	0.06 / 30.36	0.05 / 29.22
τ_2 (ns) / (%)	1.55 / 79.14	1.47 / 79.41	1.59 / 74.78	1.21 / 69.64	1.99 / 70.78
τ (ns) [a]	1.52	1.44	1.19	1.18	1.97

[a] τ = average fluorescence lifetime calculated by $\tau = \Sigma A_i\tau_i^2/\Sigma A_i\tau_i$, where A_i is the pre-exponential factor for lifetime τ_i.

Supplementary Table 12 Fluorescence lifetime data of TPO-Br powder at different temperature under air (λ_{ex} = 375 nm, λ_{em} = 434 nm).

	298 K	348 K	398 K	448 K	498 K
τ_1 (ns) / (%)	0.05 / 88.28	0.04 / 89.58	0.08 / 85.31	0.02 / 89.88	0.02 / 91.83
τ_2 (ns) / (%)	0.90 / 11.72	0.80 / 10.42	0.59 / 14.69	0.62 / 10.12	0.60 / 8.17
τ (ns) [a]	0.65	0.57	0.37	0.49	0.44

[a] τ = average fluorescence lifetime calculated by $\tau = \Sigma A_i\tau_i^2/\Sigma A_i\tau_i$, where A_i is the pre-exponential factor for lifetime τ_i.

Supplementary Table 13 Phosphorescence lifetime data of TPO-I powder at different temperature under air (λ_{ex} = 350 nm, λ_{em} = 559 nm).

	298 K	348 K	398 K	448 K	498 K
τ_1 (µs) / (%)	30.27 / 36.38	27.16 / 71.82	7.02 / 14.81	/	/
τ_2 (µs) / (%)	61.03 / 63.62	58.13 / 28.18	22.10 / 85.19	/	/
τ (µs) [a]	54.23	41.30	21.31	/	/

[a] τ = average phosphorescence lifetime calculated by $\tau = \Sigma A_i\tau_i^2/\Sigma A_i\tau_i$, where A_i is the pre-exponential factor for lifetime τ_i.

Supplementary Table 14. Phosphorescence lifetime data of TPO-Br powder at different temperature under air (λ_{ex} = 350 nm, λ_{em} = 549 nm).

	298 K	348 K	398 K	448 K	498 K
τ_1 (µs) / (%)	269 / 35.69	95 / 47.80	31 / 38.28	5 / 47.65	3 / 50.02
τ_2 (µs) / (%)	715 / 64.31	284 / 52.20	83 / 61.72	13 / 52.35	12 / 49.98
τ (µs) [a]	637	240	73	11	10

[a] τ = average phosphorescence lifetime calculated by $\tau = \Sigma A_i\tau_i^2/\Sigma A_i\tau_i$, where A_i is the pre-exponential factor for lifetime τ_i.

Supplementary Table 15 Crystallographic and structural refinement data of TPO-I and TPO-Br.

	TPO-I	TPO-Br
Empirical formula	$C_{27}H_{20}INO$	$C_{27}H_{20}BrNO$
Formula weight	501.34	454.35
Temperature (K)	293(2) K	130
Wavelength (Å)	0.71073	0.71073
Crystal system	monoclinic	monoclinic
space group	P 21/n	P 1 21/c 1
a (Å)	10.1672(10)	17.168(7)
b (Å)	18.4224(18)	10.100(4)
c (Å)	12.1232(11)	13.276(5)
α (deg)	90	90
β (deg)	99.428(3)°	109.827(6)°
γ (deg)	90	90
Volume (Å^3)	2240.1(4)	2165.5(15)
Z	4	4
Density (calculated)	1.487 Mg/m^3	1.394 Mg/m^3
Absorption coefficient	1.447 mm^{-1}	1.916 mm^{-1}
F(000)	1000	928
Crystal size	0.140 x 0.100 x 0.060 mm^3	0.165 x 0.1 x 0.01 mm^3
Theta range for data collection	2.030 to 25.499°.	2.378 to 27.739°.
Index ranges	-9<=h<=12, -22<=k<=21, -14<=l<=14	-22<=h<=21, -13<=k<=12, -17<=l<=17
Reflections collected	12769	16988
Independent reflections	4172 [R(int) = 0.0787]	5056 [R(int) = 0.1462]
Completeness to theta = 25.242°	99.8 %	99.9 %
Absorption correction	Semi-empirical from equivalents	Semi-empirical from equivalents
Max. and min. transmission	0.7456 and 0.6313	0.7456 and 0.5022
Refinement method	Full-matrix least-squares on F^2	Full-matrix least-squares on F^2
Data / restraints / parameters	4172 / 0 / 271	5056 / 0 / 271
Goodness-of-fit on F^2	1.012	0.899
Final R indices [I>2sigma(I)]	R1 = 0.0676, wR2 = 0.1266	R1= 0.0642, wR2 = 0.1464
R indices (all data)	R1 = 0.1278, wR2 = 0.1497	R1= 0.1754, wR2 = 0.1922
Extinction coefficient	n/a	n/a
Largest diff. peak and hole	0.605 and -0.447 e.Å^{-3}	1.314 and -0.849 e.Å^{-3}

Supplementary Methods

General procedure for the synthesis of TPO-X Compound **3** (0.50 g, 1.28 mmol) and phosphorus pentoxide (0.22 g, 1.54 mmol) were dissolved in dry dichloromethane (DCM) (1.0 mL) and the resulting mixture was heated under nitrogen at 180 °C for 4 h. After cooled to -20 °C, a solution of NaX (6.40 mmol) in H_2O and acetone (10 mL) was added to the reaction mixture and stirred at -20 °C for 30 min. After slowly warming to room temperature and stirring for 2 h, acetone was removed under vacuum. The solid was filtered, washed with water and purified by flash column chromatography using DCM/MeOH mixture (1:0-10:1, v/v) as eluent to afford the desired compound TPO-X.

The synthesis of TPO-I Following the general procedure for the synthesis of TPO-X, TPO-I was obtained as a yellow solid (yield: 81%). ^{1}H NMR (400 MHz, CD_3OD) δ 7.78 – 7.74 (m, 3H), 7.71 – 7.66 (m, 2H), 7.64 – 7.45 (m, 15H). ^{13}C NMR (100 MHz, CD_3OD) δ 158.86, 147.97, 134.08, 131.25, 131.11, 130.61, 130.53, 130.33, 130.11, 129.61, 129.20, 128.66, 128.56, 128.41, 126.80, 125.64, 123.91, 122.44, 119.75. HRMS (MALDI-TOF): *m/z*: $[M-I]^+$ calculated for $C_{27}H_{20}NO^+$: 374.1539; found: 374.1533.

The synthesis of TPO-Br Following the general procedure for the synthesis of TPO-X, TPO-Br was obtained as a white solid (yield: 72%). ^{1}H NMR (400 MHz, CD_3OD) δ 7.80 – 7.73 (m, 3H), 7.71 – 7.66 (m, 2H), 7.65 – 7.43 (m, 15H). ^{13}C NMR (100 MHz, CD_3OD) δ 158.83, 147.94, 134.06, 131.23, 131.08, 130.58, 130.50, 130.31, 130.08, 129.59, 129.17, 128.64, 128.53, 128.39, 126.77, 125.62, 123.88, 122.42, 119.72. HRMS (MALDI-TOF): *m/z*: $[M-Br]^+$ calculated for $C_{27}H_{20}NO^+$: 374.1539; found: 374.1557.

The synthesis of TPO-Cl Following the general procedure for the synthesis of TPO-X, TPO-Cl was obtained as a white solid (yield: 35%). ^{1}H NMR (400 MHz, CD_3OD) δ 7.78 – 7.75 (m, 3H), 7.71 – 7.66 (m, 2H), 7.64 – 7.44 (m, 15H). ^{13}C NMR (100 MHz, CD_3OD) δ 158.05, 147.06, 133.34, 130.35, 130.32, 129.73, 129.65, 129.54, 129.21, 128.85, 128.22, 127.87, 127.77, 127.61, 125.85, 124.65, 122.96, 121.52, 118.79. HRMS (MALDI-TOF): *m/z*: $[M-Cl]^+$ calculated for $C_{27}H_{20}NO^+$: 374.1539; found: 374.1579.

The synthesis of TPO-F Following the general procedure for the synthesis of TPO-X, TPO-F was obtained as a white solid (yield: 18%). ^{1}H NMR (400 MHz, CD_3OD) δ 7.81 – 7.72 (m, 3H), 7.69 – 7.66 (m, 2H), 7.64 – 7.41 (m, 15H). ^{13}C NMR (100 MHz, CD_3OD) δ 158.95, 148.06, 134.18, 131.34, 131.20, 130.70, 130.62, 130.43, 130.20, 129.71, 129.29, 128.75, 128.65, 128.51, 126.89, 125.74,

124.00, 122.54, 119.84. HRMS (MALDI-TOF): *m/z*: $[M-F]^+$ calculated for $C_{27}H_{20}NO^+$: 374.1539; found: 374.1530.

Computational Details The computational models were built from the crystal structure shown in Supplementary Figure 34. The quantum mechanics/molecular mechanics (QM/MM) theory with two-layer ONIOM method was implemented to deal with the electronic structures in crystal, where the central molecule is chosen as the active QM part and set as the high layer, while the surrounding ones are chosen as the MM part and defined as the low layer. The universal force field (UFF) was used for the MM part, and the molecules of MM part were frozen during the QM/MM geometry optimizations. On the basis of the optimized geometry of the ground state (S_0) at B3LYP/6-311G(d) level, the excitation energies and nature transition orbitals (NTOs) were calculated by using TD-DFT for electronic excited singlet and triplet states. The above results are calculated by Gaussian 09 package.[1] At the same level, the spin-orbit couplings between singlet and triplet states are given by Beijing Density Function (BDF) program.[2-4]

Preparation of nanoparticles The solids of fluorescence-active TPE-CN or RTP-active TPO-Br (2 mg) were added to the aqueous solution of 1,2-distearoyl-sn-glycero-3-phosphoethanolamine-N-[methoxy(polyethylene glycol)-2000] (DSPE-PEG, 10 mg/2 ml) under continuous sonication for 1 min, affording TPE-CN NPs and TPO-Br NPs for further imaging. Finally, the aqueous solution was filtered through a 0.22 μm polyvinylidene fluoride (PVDF) syringe-driven filter (Millipore). The size was characterized by dynamic light scattering (DLS) with a particle size analyzer (90 Plus, Brookhaven Instruments Co. USA) at a fixed angle of 90° at room temperature.

Cell imaging HeLa cells were cultured in the DMEM containing 10% FBS and antibiotics (100 units/mL penicillin and 100 g/mL streptomycin) in a 5% CO_2 humidity incubator at 37 °C. Cells were grown overnight on a 35 mm petri dish with a cover slip or a plasma-treated 25 mm round cover slip mounted at the bottom of a 35 mm petri dish with an observation window. The live cells were incubated with TPO-Br NPs at 1 mg/mL for 6 h. The labelled cells were mounted and imaged under Zeiss laser scanning confocal microscope (LSM710) and analyzed using ZEN 2009 software (Carl Zeiss). Conditions: excitation laser: 405 nm, emission collection: 500–720 nm.

***In vivo* phosphorescent imaging** All animal studies were performed in compliance with the guidelines set by the Institutional Animal Care and Use Committee, Sing Health. Female nude mice were used for *in vivo* subcutaneous imaging. For subcutaneous *in vivo* optical imaging, TPE-CN NPs and TPO-Br NPs were subcutaneously injected into the dorsal areas of mice. Then, phosphorescent

images of TPO-Br NPs solutions were acquired with the IVIS spectrum imaging system at t = 17 s after light irradiated by 365 nm handheld UV lamp (12 W) for 1 min. The UV lamp was put 1 cm above to activate nanoparticles. The IVIS spectrum imaging system was set under the bioluminescence mode with open filter and an imaging acquisition time of 17 s. Fluorescence and Phosphorescence images were analyzed by ROI analysis using the Living Image 4.0 Software. During the imaging, the mice were warmed with a heating pad under continued isoflurane anesthesia. For comparison, the *in vivo* fluorescence imaging of mice was also carried out (excitation: 430 nm).

Supplementary Discussion

Computation for TPO-I The contributions of iodine to the excited states in TPO-I are far larger than those of bromine in TPO-Br, and more outstanding heavy atom effect and charge transfer characters are exhibited (Supplementary Fig. 35). Those have more effects on the photophysical properties of TPO-I. The complete charge transfer from iodine atom to TPO core (i) forbids the electric dipole transition from the S_1 to S_0 state, which quenches the fluorescence; (ii) extremely reduces the S-T energy gap, which greatly facilitates the ISC from the S_1 to triplet states; and (iii) introduces more efficient heavy-atom effect, which further promotes more efficient ISC from the S_1 to triplet states. Thus, TPO-I is observed phosphorescent only. It is should be noted that unfortunately the calculated excitation energies of the excited states all are underestimated when compared with the experimental values owing to the defect of theoretical method.

The stability of single-molecule white light emission The stable PL spectra and CIE coordinate evidently demonstrated the good stability of the white light emission of TPO-Br film at room temperature, even at -20 ^{o}C and 40 ^{o}C. With increasing the temperature to 70 ^{o}C, the PL spectra of TPO-Br films displayed a slight increase of the ratio between phosphorescent and fluorescent intensity with the CIE coordinate changed from (0.30, 0.32) to (0.32, 0.38) during 20 days, indicating the enhancement of yellow phosphorescent emission. That is easily understood by the slow crystallization process under 70 ^{o}C.

Cell imaging and *in vivo* phosphorescent imaging TPO-Br NPs were obtained with the average size of about 250 nm as shown in Supplementary Fig. 41a. Before cell imaging, the cytotoxicity of TPO-Br NPs to live HeLa cells was demonstrated to be very low, which was suggested by the results in Supplementary Fig. 41b. Then, the luminescent imaging of HeLa cells stained by TPO-Br NPs were conducted and the luminescence was monitored by laser scanning confocal microscope. The

corresponding confocal images are shown in Supplementary Fig. 41c, where intense yellow emission is clearly observed in HeLa cells, indicating good performance in cell imaging of TPO-Br NPs.

Due to the average lifetime of TPO-Br is as long as 0.71 ms, its phosphorescence can be detected by the IVIS spectrum imaging system after UV light excitation is turned off. As shown in Supplementary Fig. 42, reported AIEgens TPE-CN and TPO-Br were utilized to fabricate fluorescent TPE-CN NPs and phosphorescent TPO-Br NPs with the help of DSPE-PEG, respectively. In Supplementary Fig. 42a, both the solutions of TPE-CN NPs and TPO-Br NPs showed fluorescence under UV light excitation (*Left*). When the UV light turned off, the solution of TPO-Br NPs exhibited phosphorescence while no phosphorescence signal was observed in TPE-CN NPs solution (*Right*). Further, we conducted the phosphorescent imaging *in vivo*, as shown in Supplementary Fig. 42b. The fluorescent imaging of living nude mouse after subcutaneous injection of TPE-CN NPs suffered from the serious autofluorescence stemmed from biological background. In sharp contrast, the living nude mouse after injection of TPO-Br NPs was clearly observed the phosphorescence signals with extremely low background due to the longer lifetime of phosphorescence than that of fluorescence. Thus the phosphorescence properties of TPO-Br make it suitable for imaging with high signal to noise *in vivo*.

Supplementary References

1. Frisch, M. J. *et al*. Gaussian, Inc.: Wallingford, CT, USA (2009).
2. Liu, W., Wang, F., Dai, D., Li, L. & Dolg, M. The Beijing Four-Component Density Functional Program Package (BDF) And Its Application to EuO, EuS, YbO and YbS. *Theor. Chem. Acc.* **96**, 75-83 (1997).
3. Liu, W., Hong, G. & Li, L. The Beijing Density Functional (BDF) Program Package: Methodologies and Applications, *J. Theor. Comput. Chem.* **2**, 257-272 (2003).
4. Hirao, K. & Ishikawa, Y. Recent Advances in Computational Chemistry, *World Scientific*, *Singapore*, **5**, 257 (2004).
